# Fatal *Ehrlichia muris eauclairensis* Infection in Liver Transplant Recipient, Minnesota, USA

**DOI:** 10.3201/eid3201.250893

**Published:** 2026-01

**Authors:** Syeda Sahra, Supavit Chesdachai, Paschalis Vergidis, William Sanchez, Bobbi S. Pritt

**Affiliations:** Mayo Clinic, Rochester, Minnesota, USA

**Keywords:** ehrlichiosis, Ehrlichia muris eauclairensis, bacteria, vector-borne infections, zoonoses, immunocompromise, ticks, liver transplant, Minnesota, United States

## Abstract

*Ehrlichia muris eauclairensis* bacterial infections can manifest with atypical and severe symptoms in immunocompromised patients. We report a fatal case of severe ehrlichiosis caused by *E. muris eauclairensis* in a liver transplant recipient in Minnesota, USA. Healthcare providers must remain vigilant about tickborne infections in endemic regions, especially among immunocompromised patients.

Ehrlichiosis is a tickborne zoonosis caused by intracellular, *Rickettsia*-like *Ehrlichia* spp., which were first described in humans in 1987 (*1*). The primary human pathogens are *E. chaffeensis*, which causes human monocytic ehrlichiosis, and *E. ewingii* and *E. muris eauclairensis* (EME), which cause granulocytic ehrlichiosis. EME, identified in 2009 in Eau Claire, Wisconsin, USA, occurs mainly in the Upper Midwest, particularly Minnesota and Wisconsin (*2*). It is transmitted by the blacklegged tick (*Ixodes scapularis*), and incidence peaks in summer (*2*). 

Clinically, ehrlichiosis manifests as an acute febrile illness, most often with fever (≈90%), headache, malaise, myalgia, and gastrointestinal symptoms (nausea, vomiting, diarrhea). Laboratory findings commonly include thrombocytopenia (≈76% of cases), leukopenia, lymphopenia, and elevated aspartate transferase (≈46% cases); rash occurs in ≈17% of cases (*3*). Neurologic symptoms such as confusion, amnesia, or seizures have been reported in ≈9% of cases, particularly in older or immunosuppressed patients, usually with unremarkable imaging (*4*). We report a fatal case of severe ehrlichiosis caused by *E. muris eauclairensis* in a liver transplant recipient in Minnesota.

In May 2025, a 52-year-old man from northern Minnesota who had liver cirrhosis and hepatocellular carcinoma underwent deceased-donor liver transplantation. Seven months later, he sought care for a 3-day history of headache, blurred vision, malaise, and throat congestion. His immunosuppression regimen included mycophenolate, sirolimus, cyclosporine, and prednisone. He had multiple episodes of glucocorticoid-resistant acute T-cell–mediated rejection treated with 4 doses of antithymocyte globulin, most recently 1 month earlier.

At the time of examination, the only notable finding was jaundice. The patient lived on a farm with tick exposure but denied known bites. Laboratory tests showed anemia, lymphopenia, thrombocytopenia, transaminitis, and hyperbilirubinemia (Table). A peripheral blood smear obtained at admission was negative for intracellular morulae. PCR tests for cytomegalovirus, Epstein-Barr virus, herpes simplex virus types 1 and 2, human herpesvirus 6, influenza, and SARS-CoV-2 were negative. Ferritin was elevated (331 µg/L). The patient was started on cefepime for neutropenic fever, but worsening headaches and confusion developed within 24 hours. Brain magnetic resonance imaging showed no acute infarcts. Empiric meningitis treatment was initiated (vancomycin, cefepime, and ampicillin). Lumbar puncture yielded clear yellow cerebrospinal fluid with 1 leukocyte, protein 17 mg/dL, and glucose 74 mg/dL. Results of cerebrospinal fluid studies, including Gram stain, HSV PCR, and a meningitis-encephalitis panel, were negative. On hospitalization day 3, a tickborne disease panel was ordered, and doxycycline (100 mg intravenously every 12 h) was initiated. By day 4, acute hypoxic respiratory failure developed, requiring intubation and intensive care unit transfer.

**Table:** Laboratory values at hospital admission for liver transplant recipient who later died of *Ehrlichia muris eauclairensis* infection, Minnesota, USA

Test (reference range)	Value
Hemoglobin, g/dL (13.2–16.6)	9.6
Platelets, × 10^9^/L (135–317)	59
Leukocytes, × 10^9^ cells/L (3.4–9.6)	1.2
Lymphocytes, × 10^9^ cells/L (0.95–3.07)	0.04
Creatinine, mg/dL (0.74–1.35)	0.53
Alanine aminotransferase, U/L (7–55)	88
Aspartate aminotransferase, U/L (8–48)	142
Total bilirubin, mg/dL (0.0–12)	18.2

On hospitalization day 5, results were received from the tickborne disease panel, a real-time PCR and DNA probe hybridization assay performed on whole blood at Mayo Clinic Laboratories (Rochester, MN, USA). The tests were negative for *Anaplasma phagocytophilum*, *E. chaffeensis*, *Babesia* spp., and *Borrelia miyamotoi* but positive for *E. muris eauclairensis*. Within 24 hours, the patient developed multiorgan failure and died.

Autopsy revealed patchy pulmonary edema, hemorrhage, hyaline membranes, and reactive pneumocytes in lung tissue (Figure, panel A). The liver showed extensive necrosis consistent with ischemic injury (Figure, panel B). Renal parenchyma demonstrated acute tubular injury with bile casts (Figure, panel C). The spleen was congested, and the heart exhibited moderate myocyte hypertrophy and mild perivascular fibrosis. No specific findings were reported from brain parenchymal tissue. Clinical findings supported severe ehrlichiosis manifesting with acute respiratory distress syndrome and multiorgan failure.

**Figure:**
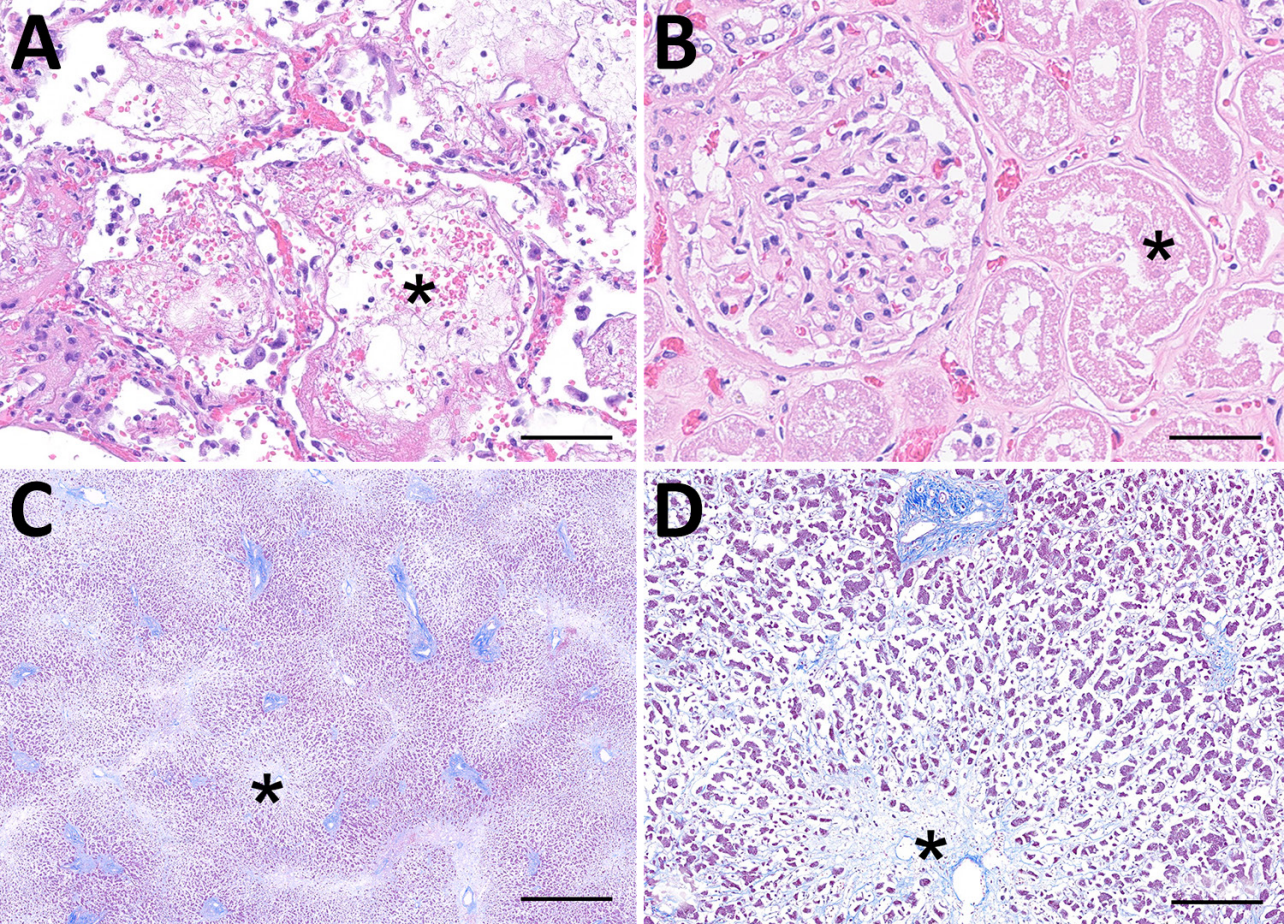
Microscopic findings from autopsy of liver transplant recipient with fatal *Ehrlichia muris eauclairensis* infection, Minnesota, USA. A) Hematoxylin and eosin stain of lung tissue shows acute diffuse alveolar damage with intraalveolar edema (asterisk) and focal hyaline membranes. Scale bar indicates 100 μm. B) Hematoxylin and eosin stain of kidney tissue shows acute tubular injury (asterisk). Scale bar indicates 50 μm. C) Masson’s trichrome stain of the liver shows prominent zone 3 necrosis (asterisk) consistent with ischemic injury. Scale bar indicates 500 μm. D) Higher magnification of panel C shows the loss of hepatocytes (asterisk) near the central vein (zone 3). Scale bar indicates 100 μm.

Diagnosis of EME relies on PCR, which is most sensitive during the first week of illness. Early serologic testing might be negative and cannot reliably differentiate EME from other species, and antibodies can persist for months to years; paired acute and convalescent serum sample testing is recommended. Treatment is doxycycline (100 mg orally or intravenously 2×/d for 7–14 days), guided by clinical response (*5*).

Immunosuppression impairs cell-mediated immunity and increases ehrlichiosis-related mortality, as demonstrated in animal models (*6*). High-risk groups include transplant recipients, asplenic patients, and patients with HIV, who can develop severe complications such as pancytopenia, renal failure, acute respiratory distress syndrome, shock, and neurologic dysfunction (*7*). In this patient, recent antithymocyte globulin therapy likely contributed to the poor outcome. That agent depletes T cells through complement-dependent lysis, activation, and apoptosis; induces B-cell apoptosis; alters leukocyte–endothelial interactions; disrupts endothelial function; and promotes regulatory and natural killer T cells (*8*).

Reported outcomes of ehrlichiosis in solid organ transplant recipients have generally been favorable; for example, 1 case of *E. chaffeensis* infection resolved despite multiorgan involvement (*9*). In a study of 75,077 US blood samples (2007–2013), EME was identified in 69 patients (0.1%), mostly in Minnesota and Wisconsin; 49 were immunocompromised, of which 13 (27%) were on immunosuppressive therapy, including 7 transplant recipients (*10*). All recovered, most after doxycycline treatment.

Clinicians should maintain a high index of suspicion for ehrlichiosis in febrile transplant recipients who have headache, altered mental status, thrombocytopenia, or transaminitis, particularly in those with tick exposure or residence in endemic areas. In this case, transaminitis and severe hyperbilirubinemia with liver allograft dysfunction complicated the diagnosis, underscoring the importance of timely tickborne disease testing. Early recognition and treatment are critical to preventing fatal outcomes.
